# Associations of clinical, psychological, and socioeconomic characteristics with nicotine dependence in smokers

**DOI:** 10.1038/s41598-021-97387-0

**Published:** 2021-09-17

**Authors:** Yun Su Sim, Seunghee Yoo, Kang-Sook Lee, Chin Kook Rhee, Young Kyoon Kim

**Affiliations:** 1 Division of Pulmonary, Allergy and Critical Care Medicine, Department of Internal Medicine, Hallym University Kangnam Sacred Heart Hospital, Seoul, Republic of Korea; 2 Health Promotion Center, The Catholic University of Korea, Seoul, Republic of Korea; 3 Department of Preventive Medicine, College of Medicine, The Catholic University of Korea, Seoul, Republic of Korea; 4 Respiratory Division of Pulmonary, Allergy and Critical Care Medicine, Department of Internal Medicine, Seoul St. Mary’s Hospital, College of Medicine, The Catholic University of Korea, Seoul, Republic of Korea

**Keywords:** Risk factors, Lifestyle modification

## Abstract

Cigarette smoking is a risk factor of mortality and morbidity from various cancerous, respiratory, and myocardial diseases. Nicotine dependence is assessed based on the degree of physical dependence. We aimed to determine the clinical, socioeconomic and psychological factors associated with the smoking status and degree of nicotine dependence of smokers. From April 2009 to September 2010, we retrospectively collected data from 17,577 subjects aged ≥ 18 years who had undergone a general health examination at a health promotion center. The instruments used included the Fagerström Tolerance Questionnaire (FTQ), Beck Depression Inventory (BDI), State-Trait Anxiety Inventory (STAI), Stress Response Inventory (SRI), and Alcohol Use Disorder Identification Test (AUDIT). Of the current smokers (*N* = 3946), 2345 (59%), 1154 (29%), and 447 (12%) had low, moderate, and high nicotine dependence, respectively. In multiple logistic analysis, predictors of high nicotine dependence were male sex (odds ratio [OR] 3.705, 95% confidence interval [CI] 1.997–6.945), older age (≥ 65 years) (OR 1.016, 95% CI 1.004–1.029), higher body mass index (BMI) (OR 1.048, 95% CI 1.018–1.078), diabetes (OR 1.870, 95% CI 1.251–2.794), single marital status (OR 1.575, 95% CI 1.186–2.092), lower education level (OR 1.887, 95% CI 1.463–2.433), and a higher stress level (OR 1.018, 95% CI 1.997–6.945). Thus, clinical, psychological, socioeconomic status including male, older age, higher BMI, diabetes, single marital status, lower education, and higher stress should be taken into consideration by promoting smoking cessation.

## Introduction

Cigarette smoking is a major risk factor for mortality and morbidity, including from head and neck cancer, lung cancer, chronic obstructive pulmonary disease (COPD), and myocardial disease^1, 2^. Smoking cessation is important for prevention of these smoking-related diseases. Unfortunately, stopping smoking is difficult due to nicotine dependence^3^. Nicotine is a psychoactive substance that affects memory, attention and performance, regulates stress responses and stabilizes mood^4^.

The relations between nicotine and mental health including depression, anxiety, stress, and alcohol dependence can be explained from various perspectives^4–10^. Depression, anxiety, and stress, can be partially relieved by a variety of neurotransmitters released after stimulation of nicotinic cholinergic receptors^4^. One of them, dopamine, signals a pleasurable experience and is critical for the reinforcing effects of nicotine^11^. These effects are increasing dependence and withdrawal symptoms of nicotine^4, 8^. The self-medication hypothesis suggests that people with psychiatric problem smoke to relieve or restore neurocognitive deficits and symptoms^6, 8^. Chronic use of nicotine might cause or exacerbate mental health problems^7, 8, 12^. Systematic review for change in mental health after smoking cessation showed that Anxiety, depression, mixed anxiety and depression, and stress significantly decreased between baseline and follow-up in quitters compared with continuing smokers^8^. The apparent relaxant effect of smoking only reflects the reversal of the tension and irritability that develop during nicotine depletion in smoker with normal moods during smoking and worsening moods between cigarettes^7^.

Previous studies have shown that high smoking rates and nicotine dependence are associated with major depression^13–22^ and anxiety^13, 15, 16, 19–25^, alcohol dependence^26^, and stress^27^. Nicotine dependence is assessed based on the degree of physical dependence to nicotine can be estimated by questionnaire^28^, which is useful for predicting the success rate of smoking cessation and provide a guide for nicotine replacement therapy^29^. Therefore, analyzing the factors related to nicotine dependence and correcting those factors will be helpful in quitting smoking. Our study was performed to determine clinical, psychological and socioeconomic factors related to nicotine dependence in smokers who underwent a health checkup (Supplementary Information 1).

## Methods

### Study subjects and design

We retrospectively collected data from 24,579 subjects aged ≥ 18 years who underwent a general health examination at the Health Promotion Center of Seoul St. Mary’s Hospital, Catholic University Hospital from April 2009 to September 2010. After excluding individuals with incomplete responses (n = 6294), and those with a cancer history (n = 503) and foreign nationals (n = 205), 17,577 subjects were included in the analysis. The Health Promotion Center serves about 15,000 people annually, providing various types of health checkups. The questionnaire survey and health examination were administered to all participants as part of a routine checkup. A separate questionnaire was not developed for this study, and the data that were originally being surveyed by all the examinees visiting the health examination center were analyzed retrospectively. The original questionnaire and the English version have been uploaded to the supporting information file. The Institutional Review Board of Seoul St. Mary’s Hospital approved the study protocol (KC11RISI0003). The patient data and questionnaire used in this study were analyzed retrospectively using the questionnaire received from the patient for the purpose of understanding health status and lifestyle guidance at the health examination center and approved as was waived by the Institutional Review Board of Seoul St. Mary’s Hospital. Patient information was anonymized and de-identified before analysis; therefore, requirements for informed consent were waived. This study was conducted in accordance with the Declaration of Helsinki.

### Variables and measures

For the smoking questionnaire, they were asked to choose one of the three answers: 'I am currently smoking’, 'I smoked in the past, but I quit’, and ‘I am currently smoking'. Those who said they smoked but quit were defined as ex-smokers, and those who said they were currently smoking were defined as current smokers. Smoker was defined current smoker and ex-smoker. Current smokers were additionally asked the age at which they started smoking, and ex-smokers were additionally asked the total period of smoking, the period of quitting, and the average amount of cigarettes smoked per day.

During the health checkup, a standard questionnaire was used to obtain information on education level (less than high school, high school graduate, college, or postgraduate), household income (< 2500, 2500–5000, 5000–7500, 7500–12,000, or > 12,000 USD/month), marital status (married, never married, divorced, or widowed), and history of comorbidities.

Smoking patients completed the Fagerström Tolerance Questionnaire (FTQ), for which a total nicotine dependence score from 0 to 11 points is generated based on the sum of the eight questionnaire items, which are variably weighted (mild = 0–3; moderate = 4–6; severe = 7–11)^28^.

Depressive symptoms were assessed using the Korean version of the self-administered 21-item Beck Depression Inventory (BDI)^30^. The validation of BDI in Korea showed significant positive internal consistency (r = 0.88)^31^.

Stress was evaluated with the Stress Response Inventory (SRI) developed by Koh et al. in 2000^32^. The SRI consists of 39 items assigned a score of 0–4 points, where higher scores indicate greater stress. The SRI used in this study is an evaluation tool developed to measure the stress of Korean with internal consistency and Cronbach's alpha for the seven subscales ranged between 0.76–0.91 and 0.97 for the total score^32^.

Anxiety symptoms were assessed with the State-Trait Anxiety Inventory (STAI), which was introduced by Spielberger in 1970 and has been translated into Korean^33^. The STAI consists of two self-report scales assessing state anxiety (unpleasant feelings and tension, with intensity levels varying according to the situation) and trait anxiety (stable personality characteristic). In Korea, Jeong-taek Kim first adapted it in 1978^33^, and a standardized study^34^ for a group of college students and a study^35^ to evaluate its application to Korea in patients with anxiety disorders were conducted.

Alcohol dependence was evaluated using the Alcohol Use Disorder Identification Test (AUDIT), a 10-item questionnaire developed by the World Health Organization to measure alcohol dependence, which has been validated internationally as a tool to screen for alcohol use disorders^36^. The cronbach's alpha was 0.82 in Korea^37^. The AUDIT evaluates the frequency of alcohol consumption, number of drinks per occasion, presence of addiction or dependence, and interference with everyday activities. Scores are classified as follows: 1–7 low-risk consumption, 8–15 hazardous or harmful alcohol consumption, 15 or more is likelihood of alcohol dependence^38^.

### Statistical analysis

Descriptive data are expressed as the mean with standard deviation (SD), and frequencies are expressed as number (%). The Chi-squared test or Fisher’s exact test was used for analyzing categorical variables, with Student’s *t* test and one-way ANOVA with Scheffé’s test used for continuous variables. Smokers’ clinical, social and psychological characteristics were compared to non-smokers and smoker using Chi-square and *t* tests. The Chi-square and *t* test were also used to compare the clinical, psychological, and social characteristics of non-smoker and ex-smoker. The effect size of the variables analyzed through the *t* test was calculated and attached to the table. ANOVA with Scheffe’s post hoc test was used to analyze clinical, psychological, and social status according to nicotine dependence. Variables significant at *p* < 0.05 in the univariate analysis, as well as sex and age, were included in the multivariate analysis. Odds ratios (ORs) with 95% confidence intervals (CIs) are reported. All *p*-values were two-sided, and *p* < 0.05 was taken to indicate statistical significance.

## Results

A total of 6399 of the 17,577 patients (36%) included in the study had a history of smoking. The demographic characteristics and mental health data of the patients are shown in Table 1. About 5% of the female subjects had a history of smoking. Overall, the smokers had a higher body mass index (BMI). However, while the BMI was higher in male smokers compared to male non-smokers, among the females the non-smokers had a higher BMI. Although hypertension and diabetes appeared to be more common in smokers, there was no significant difference between the male smokers and non-smokers, while hypertension among females was actually more prevalent in non-smokers than in smokers. With regard to marital status, there was no difference between male smokers and non-smokers, while female smokers were more likely to be unmarried than female non-smokers. The results showed that, overall, non-smokers had a lower education level and lower income, although there was no difference in income or education level according to smoking status among females. Depression, stress, and anxiety scores were higher in non-smokers among the entire subjects while alcohol dependence was higher among smokers than non-smoker. However, in each of the male and female groups, depression, anxiety, and stress scores were higher in smokers than in non-smokers.

**Table 1:** Clinical characteristics and mental health status of the smokers and non-smokers.

Variable	All participants				Males				Females			
	Smokers (*n* = 6399)	Non-smokers (*n* = 11,178)	*p*-value	Effect size	Smokers (*n* = 6048)	Non-smokers (*n* = 4774)	*p*-value	Effect size	Smokers (*n* = 351)	Non-smokers (*n* = 6404)	*p*-value	Effect size
Sex, male	6048 (95%)	4774 (43%)	< 0.001		–	–	–		–	–	–	
Age, years	45 ± 10	46 ± 11	0.527	0.10	46 ± 10	47 ± 11	< 0.001	0.13	40 ± 10	45 ± 11	< 0.001	0.44
Body mass index, kg/m^2^	24.5 ± 3.2	23.1 ± 3.2	< 0.001	0.42	24.6 ± 3.1	24.4 ± 2.9	0.001	0.07	21.6 ± 3.5	22.1 ± 3.1	0.002	0.15
Hypertension	1001 (15%)	1372 (12%)	< 0.001		984 (16%)	828 (17%)	0.137		17 (5%)	544 (9%)	0.016	
Diabetes	298 (5%)	322 (3%)	< 0.001		295 (5%)	206 (4%)	0.167		3 (1%)	116 (2%)	0.185	
Marital status, single	931 (15%)	1536(14%)	0.138		799 (13%)	603 (13%)	0.372		132 (38%)	933 (15%)	< 0.001	
Education, less than high school	924 (14%)	2577 (23%)	< 0.001		811 (13%)	632 (13%)	0.795		113 (32%)	1945 (30%)	0.470	
Income, < 2500 USD/month	718 (11%)	1569 (14%)	< 0.001		654 (11%)	602 (13%)	0.004		64 (18%)	967 (15%)	0.112	
BDI score	5.7 ± 5.6	6.5 ± 6.4	< 0.001	0.13	5.4 ± 5.2	4.5 ± 4.7	< 0.001	0.17	10.7 ± 8.3	8.0 ± 7.0	< 0.001	0.38
SRI score	21 ± 20	22 ± 21	< 0.001	0.06	20 ± 19	17 ± 17	< 0.001	0.16	34 ± 26	26 ± 23	< 0.001	0.39
State anxiety	34 ± 12	35 ± 12	< 0.001	0.09	33 ± 11	32 ± 11	< 0.001	0.10	42 ± 13	37 ± 13	< 0.001	0.40
Trait anxiety	34 ± 11	35 ± 11	< 0.001	0.14	33 ± 10	32 ± 10	< 0.001	0.08	41 ± 12	37 ± 12	< 0.001	0.29
Alcohol dependence	10 ± 6	5 ± 6	< 0.001	0.94	10 ± 7	8 ± 6	< 0.001	0.41	7 ± 7	2 ± 4	< 0.001	1.21

*BDI* Beck Depression Inventory, *SRI* Stress Response Inventory.

Data are presented as the mean (standard deviation) or number (%).

The smoker included 3946 (62%) current smokers. Ex-smokers were older and showed a higher prevalence of hypertension than current smokers. Current smokers were more likely to be single and had a lower education level and lower income than ex-smokers. Furthermore, current smokers had poorer mental health status than ex-smokers, including higher levels of depression, stress, anxiety, and alcohol dependence (Table 2).

**Table 2:** Clinical characteristics and mental health in smokers.

Variable	Current smoker (n = 3946)	Ex-smoker (n = 2453)	*p*-value	Effect size
Sex, male	3722 (94%)	2326 (95%)	0.394	
Age, years	44 ± 9	48 ± 10	< 0.001	0.45
Body mass index, kg/m^2^	24.5 ± 3.3	24.5 ± 3.0	0.755	0.01
**Co-morbid conditions**				
Hypertension	464 (12%)	537 (22%)	< 0.001	
Diabetes	170 (4.3%)	128 (5.2%)	0.093	
Marital, status, single	699 (18%)	232 (9%)	< 0.001	
Education, under high school	620 (16%)	304 (12%)	< 0.001	
Income, under 2500 USD/month	485 (12%)	233 (10%)	0.001	
Depression	6.1 ± 5.9	5.1 ± 5.1	< 0.001	0.18
Stress	22 ± 21	18 ± 18	< 0.001	0.20
**Anxiety**				
State-anxiety	34 ± 12	33 ± 12	< 0.001	0.14
Trait-anxiety	34 ± 11	33 ± 10	< 0.001	0.10
Alcohol dependence	11 ± 7	9 ± 6	< 0.001	0.27

Data are presented as mean with standard deviation or number (%).

The mean FTQ score was 3.31 ± 2.32 among current smokers. The number of current smokers with low, moderate, and high nicotine dependence was 2345 (59%), 1154 (29%), and 447 (12%), respectively. According to ANOVA, high nicotine dependence in smokers was associated with male sex, a higher BMI, diabetes, single marital status, lower education level, and lower income (Table 3).

**Table 3:** Clinical characteristics and mental health according to nicotine dependence.

Variable	Low nicotine dependence (n = 2345)	Moderate nicotine dependence (n = 1154)	High nicotine dependence (n = 447)	*p*-value
Sex, male	2184 (93%)	1103 (96%)	435 (97%)	< 0.001
Age, years	44 ± 9	44 ± 9	45 ± 9	0.143
Body mass index, kg/m^2^	24.3 ± 3.1	24.6 ± 3.7	25.0 ± 3.4	< 0.001
**Co-morbid conditions**				
Hypertension	273 (12%)	142 (12%)	49 (12%)	0.727
Diabetes	78 (3%)	55 (5%)	37 (8%)	< 0.001
Smoking, pack year	14 ± 6	20 ± 8	25 ± 7	< 0.001
Marital status, single	363 (16%)	238 (21%)	98 (22%)	< 0.001
Education, under high school	307 (13%)	205 (18%)	111 (25%)	< 0.001
Income, under 2500 USD/month	255 (11%)	158 (14%)	72 (16%)	0.002
Depression	5.4 ± 5.3	6.8 ± 6.2	7.8 ± 6.9	< 0.001
Stress	20 ± 20	24 ± 21	30 ± 26	< 0.001
**Anxiety**				
State-anxiety	33 ± 11	36 ± 12	38 ± 13	< 0.001
Trait-anxiety	33 ± 10	35 ± 11	37 ± 12	< 0.001
Alcohol dependence	10 ± 6	11 ± 7	12 ± 8	0.002

Data are presented as mean with standard deviation or number (%).

**Figure 1:**
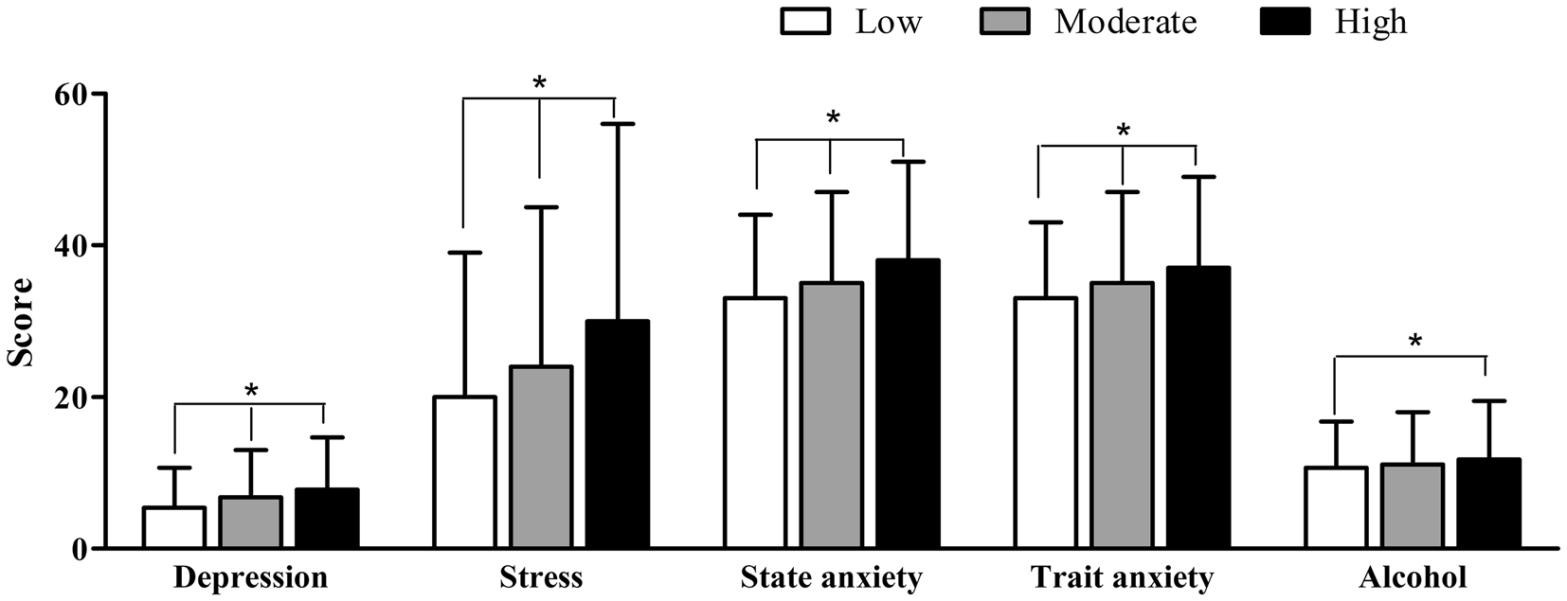
Mental health according to nicotine dependence. White bars, low nicotine dependence, Grey bars, moderate nicotine dependence, Black bars, high nicotine dependence, **┬**, standard deviation, *p < 0.05. The depression (p < 0.001), the stress score (p < 0.001), state (p < 0.001), trait (p < 0.001)—anxiety and alcohol score (p = 0.002) were higher with higher nicotine dependence. These results were also significant in the Scheffe’s post hoc test except alcohol dependence. Alcohol dependence showed a statistically significant difference only in the group with high nicotine dependence and the group with low nicotine dependence.

Worse mental health status was associated with a higher degree of nicotine dependence. The BDI (*p* < 0.001), the stress score (*p* < 0.001), state (*p* < 0.001), trait (*p* < 0.001)—anxiety and alcohol score (*p* = 0.002) were higher with higher nicotine dependence. These results were also significant in the Scheffe’s post hoc test except alcohol dependence. Alcohol dependence showed a statistically significant difference only in the group with high nicotine dependence and the group with low nicotine dependence (Table 3, Fig. 1).

In multiple logistic analyses, predictors of high nicotine dependence were male sex (OR 3.705, 95% CI 1.997–6.945), older age (OR 1.016, 95% CI 1.004–1.029), higher BMI (OR 1.048, 95% CI 1.018–1.078), diabetes (OR 1.870, 95% CI 1.251–2.794), single marital status (OR 1.575, 95% CI 1.186–2.092), lower education level (OR 1.887, 95% CI 1.463–2.433), and higher stress level (OR 1.018, 95% CI 1.997–6.945) (Table 4).

**Table 4:** Predictor of high nicotine dependence.

Variable	Univariate analysis			Multivariate analysis		
	Odd ratio	95% Confidence interval	*p*-value	Odd ratio	95% Confidence interval	*p*-value
Sex, male	2.338	1.296–4.218	0.005	3.705	1.977–6.945	< 0.001
Age, years	1.009	0.998–1.020	0.100	1.016	1.004–1.029	0.009
Body mass index, kg/m^2^	1.048	1.020–1.077	0.001	1.048	1.018–1.078	0.001
Diabetes	2.284	1.564–3.335	< 0.001	1.870	1.251–2.794	0.002
Marital status, single	1.354	1.064–1.722	0.014	1.575	1.186–2.092	0.002
Education, under high school	1.941	1.535–2.453	< 0.001	1.887	1.463–2.433	< 0.001
Income, under 2500 USD/month	1.435	1.093–1.884	0.009	1.111	0.819–1.507	0.497
Depression	1.049	1.033–1.064	< 0.001	0.999	0.975–1.023	0.926
Stress	1.018	1.014–1.022	< 0.001	1.018	1.012–1.025	< 0.001
**Anxiety**						
State-anxiety	1.027	1.018–1.035	< 0.001	1.008	0.992–1.025	0.322
Trait-anxiety	1.027	1.018–1.036	< 0.001	0.997	0.980–1.014	0.727
Alcohol dependence	1.024	1.009–1.039	0.002	1.013	0.998–1.028	0.098

## Discussion

In our study, the mental health of non-smokers was lower than that of smokers. However, when comparing non-smokers and smokers by gender by gender, smokers' mental health scores were poor in both groups. In the smoker group, 95% of males and 5% of females were male, whereas in non-smokers, 47% of females were more than 43% of males. The mental health score of women was very low compared to men in our study. Therefore, it is considered to be a bias caused by the high ratio of females to non-smokers.

This study showed that mental health score including depression, anxiety, alcohol dependence, and stress had statistically significant odd ratio in univariated analysis according to high nicotine dependence. In multivariated analysis related to high nicotine dependence, only stress had a statistically significant OR value. Depression, anxiety, and stress, can be partially relieved by nicotine^4^. These effects are increasing dependence and withdrawal symptoms^4, 8^. The self-medication hypothesis suggests that people with psychiatric problem use nicotine to relieve or restore neurocognitive deficits and symptoms^6, 8^. However, person with high nicotine dependence have progressively poor mental health while using nicotine chronically^7, 8, 12^. Therefore, causal relationship between high nicotine dependence and poor mental health is difficult to clearly distinguish. However, since high nicotine dependence and stress had a statistically significant correlation in multivariate analysis in our study, assessing the stress score in patients with high nicotine dependence can be an important reference for determining the patient’s condition and guiding smoking cessation.

Predictors of high nicotine dependence included male sex, older age, higher BMI, diabetes, single marital status, lower education level, and higher stress levels.

Another risk factor of nicotine dependence in our study was males, but Breslau et al.^39^ reported no difference in nicotine dependence according to sex. However, Two Singaporean studies published in 2012 and 2019 showed that nicotine dependence was higher in male^40, 41^. In our study, nicotine dependence was higher with increasing age, whereas in Singapore study, nicotine dependence was higher in younger age groups^40, 41^. The severity of nicotine dependence in a middle-aged group (45–64 years old) was higher than in younger (< 45 years) and older age groups (≥ 65 years old) in another study^42^. The relationship between age, gender, and nicotine dependence requires additional research involving smokers of various races, genders, and age groups.

Nicotine dependence was more severe in smokers with a higher BMI in the present study. The study about Mendelian randomization analyses of the effects of BMI on smoking behavior indicate that higher BMI causally influences lifetime smoking, smoking initiation, smoking heaviness and also DNA methylation at the aryl-hydrocarbon receptor repressor locus^43^. Those results suggest that there may be bidirectional causal effects between smoking phenotypes and BMI, and that these may act in opposing directions^43^.

In a previous study of depression and anxiety in relation to smoking^44^, there was no significant difference in nicotine dependence between non-diabetic and diabetic smokers. However, in our study, nicotine dependence was high in diabetic patients. The study about rodent model of diabetes showed that both enhancing nicotine reward and withdrawal implies that the strong behavioral effects of nicotine promote tobacco use in persons with metabolic disorders, such as diabetes^45^. Recent animal study suggests that TCF7L2 regulates the stimulatory actions of nicotine on a habenula–pancreas axis that links the addictive properties of nicotine to its diabetes-promoting actions^46^. Another study suggests that diabetes induces a disruption in insulin signaling that leads to a suppression of dopamine systems in the mesolimbic reward pathway but, rewarding effects of nicotine promote tobacco use in persons with diabetes^47^. Smoking cessation is very important in diabetic patients, because the incidence of vascular disease is higher than in non-diabetic subjects^48^. Therefore, we should evaluate the nicotine dependence and to quit smoking in smokers with diabetes.

In a previous study of nicotine dependence related to socioeconomic status and marital status^49^, a low education level was related to high nicotine dependence, but there was no difference in nicotine dependence between subjects living without a spouse and those who were married. In the present study, nicotine dependence tended to be higher in unmarried individuals, and in those with a lower education level. These data support the hypothesis that nicotine dependence is stronger among smokers with a lower education level or unmarried status. Current smokers were younger and more likely to be single and had a lower education level and lower income than ex-smokers. With regard to measures of mental health, levels of depression, stress, anxiety, and alcohol dependence were all higher in current smokers than ex-smokers. In another study^49^, lower education level was related to the likelihood of smoking, but marriage status and income were not. However, the cross-sectional analysis conducted in the present study suggested a relationship between poor socioeconomic status and smoking; the marital status may also have an effect on smoking likelihood. Therefore, we suggest that further research on psychological factors, socioeconomic factors, and nicotine dependence will lead to clear consequences of these factors by cohort studies.

This study had several limitations. First, there was a problem with the study design. Since the ratios of males and females were not similar, gender bias existed. Therefore, since most smokers are male, the comparative analysis of current smokers and former smokers has a research limit that can be interpreted as being limited to males. In addition, it was difficult to investigate the mental health related co-morbidity in a retrospective study, only cancer patients were excluded among patients with various co-morbidities. Second, this study was performed in subjects who underwent medical health examinations; such subjects may have made more efforts to quit smoking, or pay more attention to mental health issues, compared to the general population. Third, we used self-report measures of cigarette smoking and nicotine dependence, stress, depression, anxiety, and alcohol dependence, which could be subject to recall bias.

Levels of depression, anxiety, stress, and alcohol dependence tended to be higher in participants with high nicotine dependence in univariate analysis, but only stress was statistically significant in multivariate analysis. Thus, evaluation for the stress score in patients with high nicotine dependence can be an important reference for guiding smoking cessation. And male sex, older age, higher BMI, diabetes, single marital status, and a lower education level related with high nicotine dependence. Therefore, in patients with these clinical, psychological, and socioeconomic status, evaluation of nicotine dependence will be necessary when anti-smoking treatment is performed. Furthermore, a longitudinal cohort study is needed to clearly identify the causal relationship between nicotine dependence and clinical, psychological, and socioeconomic factors.

## Supplementary Information

Supplementary Information 1.

Supplementary Information 2.
