# Supplementary material for: Associations of clinical, psychological, and socioeconomic characteristics with nicotine dependence in smokers
Source: Sci Rep. 2021 Sep 17;11:18544. doi: 10.1038/s41598-021-97387-0 (PMC8448893; doi:10.1038/s41598-021-97387-0)
Supplement: Supplementary file 1 — Supplementary Information 1. [file 41598_2021_97387_MOESM1_ESM.docx]

This is a question about social history

1. Your marital status is ?

□ single □ Bereavement □ matrimony □ 기타

2. What is your final education level?

□ Under high school graduation □ College graduation □ Graduating from high school or attending college or dropping out □ Graduate school or higher

3. What is your household's average monthly income??

< 2,500,000, 2,500,000–5,000,000, 5,000,000-7,500,000, 7,500,000–12,000,000, or > 12,000,000 USD/month

This is a question about stress..

Please indicate how much you have experienced the following questions over the past week, one for each in the appropriate box. (Example: not at all, slightly, somewhat, quite, quite, very).

1. There are many mistakes in work.

2. I hate to say.

3. The chest is stuffy.

4. I am angry.

5. I am restless.

6. I cannot digest.

7. My stomach hurts.

8. I want to scream.

9. A sigh comes out.

10. I am dizzy.

11. Everything is bothersome.

12. I have misunderstandings.

13. I feel tired easily.

14. My whole body loses strength.

15. I lost my confidence.

16. I'm nervous.

17. My body is shaking.

18. I want to hit someone.

19. My motivation fell.

20. I want to cry.

21. My nerves became sharp.

22. I have no prospects for what I do.

23. I am blank.

24. I hate someone.

25. I can't escape from one thought.

26. My voice grew.

27. I feel like I am in a hurry or running out of work.

28. The behavior has become rough. (Reckless driving, swear words, struggles, etc.).

29. I want to break something.

30. The words disappeared.

31. My head is heavy or hurt.

32. My heart is pounding.

33. I want to kill someone.

34. My face is red or hot.

35. It's boring.

36. Impatience.

37. My facial expression became hard.

38. I am a useless person.

39. I don't want to move.

This is a question about the depression scale (Beck Depression Inventory; BDI)

Think about your mood and condition over the past two weeks, and mark only one sentence from the 22 question boxes that best describes it.

1.

I do not feel sad.

I feel sad

I am sad all the time and I can't snap out of it.

I am so sad and unhappy that I can't stand it.

2.

I am not particularly discouraged about the future.

I feel discouraged about the future.

I feel I have nothing to look forward to.

I feel the future is hopeless and that things cannot improve.

3.

I do not feel like a failure.

I feel I have failed more than the average person.

As I look back on my life, all I can see is a lot of failures.

I feel I am a complete failure as a person.

4.

I get as much satisfaction out of things as I used to.

I don't enjoy things the way I used to.

I don't get real satisfaction out of anything anymore.

I am dissatisfied or bored with everything.

5.

I don't feel particularly guilty

I feel guilty a good part of the time.

I feel quite guilty most of the time.

I feel guilty all of the time.

6.

I don't feel I am being punished.

I feel I may be punished.

I expect to be punished.

I feel I am being punished.

7.

I don't feel disappointed in myself.

I am disappointed in myself.

I am disgusted with myself.

I hate myself.

8.

I don't feel I am any worse than anybody else.

I am critical of myself for my weaknesses or mistakes.

I blame myself all the time for my faults.

I blame myself for everything bad that happens.

9.

I don't have any thoughts of killing myself.

I have thoughts of killing myself, but I would not carry them out.

I would like to kill myself.

I would kill myself if I had the chance.

10.

I don't cry any more than usual.

I cry more now than I used to.

I cry all the time now.

I used to be able to cry, but now I can't cry even though I want to.

11.

I am no more irritated by things than I ever was.

I am slightly more irritated now than usual.

I am quite annoyed or irritated a good deal of the time.

I feel irritated all the time.

12.

I have not lost interest in other people.

I am less interested in other people than I used to be.

I have lost most of my interest in other people.

I have lost all of my interest in other people.

13.

I make decisions about as well as I ever could.

I put off making decisions more than I used to.

I have greater difficulty in making decisions more than I used to.

I can't make decisions at all anymore.

14.

I don't feel that I look any worse than I used to.

I am worried that I am looking old or unattractive.

I feel there are permanent changes in my appearance that make me look unattractive

I believe that I look ugly.

15.

I can work about as well as before.

It takes an extra effort to get started at doing something.

I have to push myself very hard to do anything.

I can't do any work at all.

16.

I can sleep as well as usual.

I don't sleep as well as I used to.

I wake up 1-2 hours earlier than usual and find it hard to get back to sleep.

I wake up several hours earlier than I used to and cannot get back to sleep.

17.

I don't get more tired than usual.

I get tired more easily than I used to.

I get tired from doing almost anything.

I am too tired to do anything.

18.

My appetite is no worse than usual.

My appetite is not as good as it used to be.

My appetite is much worse now.

I have no appetite at all anymore.

19.

I haven't lost much weight, if any, lately.

I have lost more than five pounds.

I have lost more than ten pounds.

I have lost more than fifteen pounds.

20.

I am no more worried about my health than usual.

I am worried about physical problems like aches, pains, upset stomach, or constipation.

I am very worried about physical problems and it's hard to think of much else.

I am so worried about my physical problems that I cannot think of anything else.

21.

I have not noticed any recent change in my interest in sex.

I am less interested in sex than I used to be.

I have almost no interest in sex.

I have lost interest in sex completely.

INTERPRETING THE BECK DEPRESSION INVENTO

3. 상태-특성 불안 척도(STATE-TRAIT ANXIETY INVENTORY)

State Anxiety: At the moment, please indicate one for each question that best describes your current state of feeling. (Example: not at all, slightly, somewhat, quite, quite, very much.)나는 마음이 차분하다.

1. I am calm.

2. I am reassuring.

3. I am nervous.

4. I regret and regret.

5. I am at ease.

6. I'm embarrassed and don't know what to do.

7. I am worried that there will be unhappiness in the future.

8. I am relieved.

9. I am anxious.

10. I feel comfortable.

11. I am confident.

12. I am annoyed.

13. I am nervous.

14. I am extremely nervous.

15. My heart is relaxed and warm.

16. I am satisfied.

17. I am worried.

18. I am excited and have no idea what to do.

19. I am happy.

20. I feel good.

Trait Anxiety: Please indicate one for each question that best describes the condition you are feeling in a normal situation, not right now. In some cases, you may be asked questions like the ones above. (Example: not at all, slightly, somewhat, quite, quite, very much.)

21. I feel good

22. I get tired easily.

23. I feel like crying.

24. I want to be happy like everyone else.

25. I fail because I can't make up my mind quickly.

26. I am relieved.

27. I am calm and calm.

28. I don't think I can overcome too many difficult problems.

29. I am so worried about trivial things.

30. I am happy.

31. I find it hard to do anything.

32. I lack confidence.

33. I am reassuring.

34. I try to avoid crises or difficulties.

35. I am depressed.

36. I am satisfied.

37. Trivial thoughts bother me.

38. I am overly sensitive to disappointment that I cannot erase it from my mind.

39. I am a good person.

40. I am nervous or don't know what to do if I just think about my worries or concerns these days..

This is an evaluation item for alcohol dependence.

How often do you have a drink containing alcohol?

□ Never □ Monthly or less □ 2 to 4 times a month □ 2 to 3 times a week □ 4 or more times a week

How many drinks containing alcohol do you have on a typical day when you are drinking?

□ 1 or 2 □ 3 or 4 □ 5 or 6 □ 7,8 or 9 □ 10 or more

How often do you have six or more drinks on one occasion?

□ Never □ Less than monthly □ Monthly □ Weekly □ Daily or almost daily

How often during the last year have you found that you were not able to stop drinking once you had started?

□ Never □ Less than monthly □ Monthly □ Weekly □ Daily or almost daily

How often during the last year have you failed to do what was normally expected from you because of drinking?

□ Never □ Less than monthly □ Monthly □ Weekly □ Daily or almost daily

How often during the last year have you needed a first drink in the morning to get yourself going after a heavy drinking session?

□ Never □ Less than monthly □ Monthly □ Weekly □ Daily or almost daily

How often during the last year have you had a feeling of guilt or remorse after drinking??

□ Never □ Less than monthly □ Monthly □ Weekly □ Daily or almost daily

How often during the last year have you be enunable to remember what happened the night before because you had been drinking?

□ Never □ Less than monthly □ Monthly □ Weekly □ Daily or almost daily

Have you or someone else been injured as a result of your drinking?

□ No □ Yes, but not in the last year. □ Yes, during the last year.

Has a relative or friend or a doctor or another health worker been concerned about your drink-ing or suggested you cut down?

□ No □ Yes, but not in the last year. □ Yes, during the last year

This is a question about smoking habits

1.What is the first time you smoke after waking up in the morning?

□ 30 minutes later □ Within 30 minutes

2. Are you having trouble withholding cigarettes in a non-smoking place such as a library, theater or hospital?

□ yes □ no

3. At what time of day are you most satisfied with the cigarettes you smoke?

□ First cigarette smoked after waking up in the morning □ Cigarettes smoked after that

4. How much do you smoke a day?

□ 1-15 □ 16-24 □ 25

5. Are there more cigarettes you smoke in the morning than you smoke in the afternoon?

□ yes □ no

6. Do you smoke even when you are sick enough to lie down?

□ yes □ no

7. How much nicotine is in the cigarettes you smoke?

□ mild □ average □ poisonous

8. Do you drink deeply when you smoke?

□ never □ sometimes □ everytime
