# Supplementary material for: Associations of clinical, psychological, and socioeconomic characteristics with nicotine dependence in smokers
Source: Sci Rep. 2021 Sep 17;11:18544. doi: 10.1038/s41598-021-97387-0 (PMC8448893; doi:10.1038/s41598-021-97387-0)
Supplement: Supplementary file 2 — Supplementary Information 2. [file 41598_2021_97387_MOESM2_ESM.docx]

사회력에 대한 질문입니다

1. 귀하의 결혼 상태는?

□ 미혼 □ 사별 □ 기혼 □ 기타

2. 귀하의 최종학력은 무엇입니까?

□ 고등학교 졸업 미만 □ 대학 졸업 □ 고등학교 졸업 또는 대학 재학, 중퇴 □ 대학원 이상

3. 귀하의 직업은 무엇입니까?

□ 최고 경영자 □ 상업, 유통, 운송 및 기타 서비스업 □ 대기업 임원 □ 농업, 수산업, 임업, 광업 □ 부장급 이상 관리자 □ 제조, 건설업 □ 사무직, 관리 행정직 □ 금융업 □ 고위공직자 □ 경찰관 □ 입법의원 □ 소방관 □ 공무원 □ 문화예술가 □ 교육자 □ 운동선수 □ 전문직(학자, 언론, 컴퓨터관련 포함) □ 군인 □ 법조인 □ 학생 □ 의료인 □ 퇴직/무직/기타 □ 종교인

4. 귀 가정의 월 평균수입은 어떠하십니까?

□ 300만원 이하 □ 300~600만원 □ 600~900만원 □ 900~1500만원 □ 1500만원 이상

3⃞ 스트레스에 관한 질문입니다.

1. 스트레스반응에 대한 질문입니다.

지난 일주일 동안 아래의 문항을 어느 정도 경험했는지 해당되는 칸에 각각 하나씩 표시하여 주십시오. (보기: 전혀 그렇지 않다, 약간 그렇다, 웬만큼 그렇다, 상당히 그렇다, 아주 그렇다).

1. 일에 실수가 많다.

2. 말하기 싫다.

3. 가슴이 답답하다.

4. 화가 난다.

5. 안절부절 못한다.

6. 소화가 안 된다.

7. 배가 아프다.

8. 소리를 지르고 싶다.

9. 한숨이 나온다.

10. 어지럽다.

11. 만사가 귀찮다.

12. 잡념이 생긴다.

13. 쉽게 피로를 느낀다.

14. 온몸에 힘이 빠진다.

15. 자신감을 잃었다.

16. 긴장된다.

17. 몸이 떨린다.

18. 누군가를 때리고 싶다.

19. 의욕이 떨어졌다.

20. 울고 싶다.

21. 신경이 날카로워졌다.

22. 내가 하는 일에 전망이 없다.

23. 멍하게 있다.

24. 누군가를 미워한다.

25. 한 가지 생각에서 헤어나지 못한다.

26. 목소리가 커졌다.

27. 마음이 급해지거나 일에 쫓기는 느낌이다.

28. 행동이 거칠어졌다. (난폭 운전, 욕설, 몸싸움 등).

29. 무엇인가를 부수고 싶다.

30. 말이 없어졌다.

31. 머리가 무겁거나 아프다.

32. 가슴이 두근거린다.

33. 누군가를 죽이고 싶다.

34. 얼굴이 붉어지거나 화끈거린다.

35. 지루하다.

36. 참을성이 없다.

37. 얼굴 표정이 굳어졌다.

38. 나는 아무 쓸모가 없는 사람이다.

39. 움직이기 싫다.

우울 척도에 대한 질문입니다 (Beck Depression Inventory; BDI)

지난 2주일 간 당신의 기분과 상태를 생각해 보시고, 22가지 질문 박스에서 이를 가장 잘

설명하는 문장 각각 1개씩만 골라 표시해 주십시오.

1

□ 나는 슬프지 않다.

□ 나는 슬프다.

□ 나는 항상 슬퍼서 그것을 떨쳐버릴 수 없다.

□ 나는 너무나 슬프고 불행해서 도저히 견딜 수 없다.

2

□ 나는 앞날에 대해서 별로 낙심하지 않는다.

□ 나는 앞날에 대해서 비관적인 느낌이 든다.

□ 나는 앞날에 대해 기대할 것이 아무 것도 없다고 느낀다.

□ 나의 앞날은 아주 절망적이고 나아질 가망이 없다고 느낀다.

3

□ 나는 실패자라고 느끼지 않는다.

□ 나는 보통사람들보다 더 많이 실패한 것 같다.

□ 내가 살아온 과거를 뒤돌아보면, 생각나는 것은 실패뿐이다.

□ 나는 인간으로서 완전한 실패자인 것 같다.

4

□ 나는 전과 같이 일상생활에 만족하고 있다.

□ 나의 일상생활은 전처럼 즐겁지 않다.

□ 나는 더 이상 어떤 것에서도 참된 만족을 얻지 못한다.

□ 나는 모든 것이 다 불만스럽고 지겹다.

5

□ 나는 특별히 죄책감을 느끼지 않는다.

□ 나는 죄책감을 느낄 때가 많다.

□ 나는 거의 언제나 죄책감을 느낀다.

□ 나는 항상 언제나 죄책감을 느낀다.

6

□ 나는 벌을 받고 있다고 느끼지 않는다.

□ 나는 아마 벌을 받을 것 같다.

□ 나는 벌을 받아야 한다고 느낀다.

□ 나는 지금 벌을 받고 있다고 느낀다.

7

□ 나는 나 자신에게 실망하지 않는다.

□ 나는 나 자신에게 실망하고 있다.

□ 나는 나 자신을 혐오스럽다.

□ 나는 나 자신을 증오한다.

8

□ 내가 다른 사람보다 못한 것 같지는 않다.

□ 나는 나의 약점이나 실수에 대해서 자신을 탓한다.

□ 내가 한 일이 잘못 되었을 때는 언제나 나를 탓한다.

□ 일어나는 모든 나쁜 일들은 다 내 탓이다.

9

□ 나는 자살 같은 것은 생각하지 않는다.

□ 나는 자살 할 생각은 하고 있으나, 실제로 하지는 않을 것이다.

□ 나는 자살하고 싶다.

□ 나는 기회만 있으면 자살하겠다.

10

□ 나는 평소보다 더 울지는 않는다.

□ 나는 전보다 더 많이 운다.

□ 나는 요즈음 항상 운다.

□ 나는 전에는 울고 싶을 때 울 수 있었지만, 요즈음은 울래야 울 기력조차 없다.

11

□ 나는 요즈음 평소보다 더 짜증을 내는 편은 아니다.

□ 나는 전보다 더 쉽게 짜증이 나고 귀찮아진다.

□ 나는 요즈음 항상 짜증스럽다.

□ 전에는 짜증스럽던 일에 요즈음은 너무 지쳐서 짜증조차 나지 않는다.

12

□ 나는 다른 사람들에 대한 관심을 잃지 않고 있다.

□ 나는 전보다 다른 사람들에 대한 관심이 줄었다.

□ 나는 다른 사람들에 대한 관심이 거의 없어졌다.

□ 나는 다름 사람들에 대한 관심이 없어졌다.

13

□ 나는 평소처럼 결정을 잘 내린다.

□ 나는 결정을 내리는 때가 전보다 더 많다.

□ 나는 전에 비해 결정 내리는 데에 더 큰 어려움을 느낀다.

□ 나는 더 이상 아무 결정도 내릴 수가 없다.

14

□ 나는 전보다 내 모습이 더 나빠졌다고 느끼지 않는다.

□ 나는 나이 들어 보이거나 매력 없어 보일까 봐 걱정한다.

□ 나는 내 모습이 매력 없게 변해 버렸다고 느낀다.

□ 나는 내가 추하게 보인다고 믿는다.

15

□ 나는 전처럼 일을 할 수 있다.

□ 어떤 일을 시작하려면 특별히 더 많은 노력이 든다.

□ 무슨 일이든 하려면 나 자신을 매우 심하게 채찍질해야만 한다.

□ 나는 전혀 아무 일도 할 수가 없다.

16

□ 나는 평소처럼 잠을 잘 수 있다.

□ 나는 전처럼 잠을 자지 못한다.

□ 나는 전보다 한 두 시간 일찍 깨고 다시 잠들기 어렵다.

□ 나는 전혀 아무 일도 할 수가 없다

17

□ 나는 평소보다 더 피곤하지는 않다.

□ 나는 전보다 더 쉽게 피곤해진다.

□ 나는 무엇을 해도 언제나 피곤해진다.

□ 나는 너무나 피곤해서 아무 일도 할 수 없다.

18

□ 내 식욕은 평소와 다름없다.

□ 나는 요즈음 전보다 식욕이 좋지 않다.

□ 나는 요즈음 식욕이 많이 떨어졌다.

□ 요즈음에는 전혀 식욕이 없다.

19

□ 요즈음 체중이 별로 줄지 않았다.

□ 전보다 몸무게가 2㎏가량 줄었다.

□ 전보다 몸무게가 5㎏가량 줄었다.

□ 전보다 몸무게가 7㎏가량 줄었다.

20

나는 현재 음식 조절로 체중을 줄이고 있는 중이다.

□ 예 □ 아니오

21

□ 나는 건강에 대해 전보다 더 염려하고 있지는 않다.

□ 나는 여러 가지 통증, 소화불량, 변비 등과 같은 신체적인 문제로 걱정하고 있다.

□ 나는 건강이 매우 염려되어 다른 일은 생각하기 힘들다.

□ 나는 건강이 너무 염려되어 다른 일은 아무 것도 생각할 수 없다.

22

□ 나는 요즈음 성(Sex)에 대한 관심에 별다른 변화가 있는 것 같지는 않다.

□ 나는 전보다 성(Sex)에 대한 관심이 줄었다.

□ 나는 전보다 성(Sex)에 대한 관심이 상당히 줄었다.

□ 나는 성(Sex)에 대한 관심을 완전히 잃었다.

3. 상태-특성 불안 척도(STATE-TRAIT ANXIETY INVENTORY)

상태불안 : 바로 지금, 현재 느낌의 상태를 가장 잘 표현해 주는 경우를 각 질문에 대해 하나씩 표시해 주십시오. (보기: 전혀 그렇지 않다, 약간 그렇다, 웬만큼 그렇다, 상당히 그렇다, 아주 그렇다.)

1. 나는 마음이 차분하다.
2. 나는 마음이 든든하다.
3. 나는 긴장되어 있다.
4. 나는 후회스럽고 서운하다.
5. 나는 마음이 편하다.
6. 나는 당황해서 어찌할 바를 모르겠다.
7. 나는 앞으로 불행이 있을까봐 걱정하고 있다.
8. 나는 마음이 놓인다.
9. 나는 불안하다.
10. 나는 편하게 느낀다.
11. 나는 자신감이 있다.
12. 나는 짜증스럽다.
13. 나는 마음이 조마조마하다.
14. 나는 극도로 긴장되어 있다.
15. 내 마음은 긴장이 풀려 푸근하다.
16. 나는 만족스럽다.
17. 나는 걱정하고 있다.
18. 나는 흥분되어 어쩔 줄 모르겠다.
19. 나는 즐겁다.
20. 나는 기분이 좋다.

특성불안 : 지금 당장이 아닌, 평소 일반적인 상황에서 당신이 느끼는 상태를 가장 잘 표현해 주는 경우를 각 질문에 대해 하나씩 표시해 주십시오. 위의 질문과 같은 질문은 묻는 경우도 있습니다. (보기: 전혀 그렇지 않다, 약간 그렇다, 웬만큼 그렇다, 상당히 그렇다, 아주 그렇다.)

1. 나는 기분이 좋다
2. 나는 쉽게 피로해진다.
3. 나는 울고 싶은 심정이다.
4. 나도 다른 사람들처럼 행복했으면 한다.
5. 나는 마음을 빨리 정하지 못해서 실패를 한다.
6. 나는 마음이 놓인다.
7. 나는 차분하고 침착하다.
8. 나는 너무 많은 어려운 문제가 밀어 닥쳐서 극복할 수 없을 것 같다.
9. 나는 하찮은 일에 너무 걱정을 한다.
10. 나는 행복하다.
11. 나는 무슨 일이건 힘들게 생각한다.
12. 나는 자신감이 부족하다.
13. 나는 마음이 든든하다.
14. 나는 위기나 어려움을 피하려고 애쓴다.
15. 나는 울적하다.
16. 나는 만족스럽다.
17. 사소한 생각이 나를 괴롭힌다.
18. 나는 실망을 지나치게 예민하게 받아들이기 때문에 머릿속에서 지워버릴 수가 없다.
19. 나는 착실한 사람이다.
20. 나는 요즈음의 걱정이나 관심거리를 생각만 하면 긴장되거나 어찌할 바를 모른다.

술 의존도 평가 항목입니다.

술을 마시는 횟수는 어느 정도 입니까?

□ 전혀 안 마심 □ 월 1회 미만 □ 월 2~4회 □ 주 2~3회 □ 주 4회 이상

술을 마시는 날은 보통 몇 잔을 마십니까?

□ 한 두 잔 □ 3~4잔 □ 5~6잔 □ 7~9잔 □ 10잔 이상

한 번의 술좌석에서 6잔 이상을 마시는 횟수는 어느 정도입니까?

□ 전혀 없다 □ 월 1회 미만 □ 월 1회 □ 주 1회 □ 거의 매일

지난 1년간 술을 마시기 시작하여 자제가 안 된 적이 있습니까?

□ 전혀 없다 □ 월 1회 미만 □ 월 1회 □ 주 1회 □ 거의 매일

지난 1년간 음주 때문에 일상생활에 지장을 받은 적이 있습니까?

□ 전혀 없다 □ 월 1회 미만 □ 월 1회 □ 주 1회 □ 거의 매일

지난 1년간 과음 후 다음날 아침 정신을 차리기 위해 해장술을 마신적이 있습니까?

□ 전혀 없다 □ 월 1회 미만 □ 월 1회 □ 주 1회 □ 거의 매일

지난 1년간 음주 후 술을 마신 것에 대해 후회한 적이 있습니까?

□ 전혀 없다 □ 월 1회 미만 □ 월 1회 □ 주 1회 □ 거의 매일

지난 1년간 술이 깬 후에 취중의 일을 기억할 수 없었던 적이 있습니까?

□ 전혀 없다 □ 월 1회 미만 □ 월 1회 □ 주 1회 □ 거의 매일

음주로 인해 본인이 다치거나 또는 가족이나 타인이 다친 적이 있습니까?

□ 없었다 □ 있지만 지난 1년간에는 없었다. □ 지난 1년간에 있었다.

가족, 의사가 음주에 대해 걱정을 하거나 또는 술을 끊거나 줄이라는 권고를 한 적이 있습니까?

□ 없었다 □ 있지만 지난 1년간에는 없었다. □ 지난 1년간에 있었다.

흡연 습관에 관한 질문입니다

1. 아침에 일어난 후 처음 담배를 피우는 시간은 ?

□ 30분 후 □ 30분 이내

2. 귀하는 금연 장소인 도서관, 극장 또는 병원 등에서 담배를 참는데 어려움을 느끼십니까?

□ 예 □ 아니오

3. 하루 중 어느 때 피우는 담배가 가장 만족스럽습니까?

□ 일어나서 처음 피우는 담배 □ 그 이후에 피우는 담배

4. 하루에 얼마나 피우십니까?

□ 1-15 개피 □ 16-24 개피 □ 25 개피

5. 오전에 피우는 담배가 오후에 피우는 것보다 더 많습니까?

□ 예 □ 아니오

6. 귀하는 누워 지내야 할 정도로 아플 때도 담배를 피우십니까?

□ 예 □ 아니오

7. 귀하가 피우는 담배는 니코틴 함량이 어느 정도 입니까?

□ 순하다 □ 보통이다 □ 독하다

8. 당신은 담배를 피울 때 깊숙이 들여 마십니까?

□ 전혀 그렇지 않다 □ 때때로 그렇다 □ 항상 그렇다
